# The pathogenesis, diagnosis and management of congenital dyserythropoietic anaemia type I

**DOI:** 10.1111/bjh.15817

**Published:** 2019-03-05

**Authors:** Noémi B. A. Roy, Christian Babbs

**Affiliations:** ^1^ MRC Molecular Haematology Unit MRC Weatherall Institute of Molecular Medicine University of Oxford Oxford UK; ^2^ BRC Blood Theme and BRC/NHS Translational Molecular Diagnostics Centre John Radcliffe Hospital Oxford UK; ^3^ Oxford University Hospitals NHS Foundation Trust John Radcliffe Hospital Oxford UK

**Keywords:** congenital dyserythropoetic anaemia, anaemia, dyserythropoiesis, erythropoiesis, clinical haematology

## Abstract

Congenital dyserythropoietic anaemia type I (CDA‐I) is one of a heterogeneous group of inherited anaemias characterised by ineffective erythropoiesis. CDA‐I is caused by bi‐allelic mutations in either *CDAN1* or *C15orf41* and, to date, 56 causative mutations have been documented. The diagnostic pathway is reviewed and the utility of genetic testing in reducing the time taken to reach an accurate molecular diagnosis and avoiding bone marrow aspiration, where possible, is described. The management of CDA‐I patients is discussed, highlighting both general and specific measures which impact on disease progression. The use of interferon alpha and careful management of iron overload are reviewed and suggest the most favourable outcomes are achieved when CDA‐I patients are managed with a holistic and multidisciplinary approach. Finally, the current understanding of the molecular and cellular pathogenesis of CDA‐I is presented, highlighting critical questions likely to lead to improved therapy for this disease.

Congenital dyserythropoietic anaemia type I (CDA‐I) (Online Mendelian Inheritance in Man [OMIM] entry: 224120; Orphanet: D64.4 and DCS‐10) is one of a heterogeneous group of disorders termed the congenital dyserythropoietic anaemias (CDAs). Unlike other rare anaemias, the CDAs are characterised by ineffective erythropoiesis and morphological abnormalities of erythroblasts. Other haematopoietic lineages are unaffected and there is a haemolytic component. Dyserythropoiesis is defined as the presence of erythroblast abnormalities indicative of aberrant proliferation or differentiation (Crookston *et al*, 1966). The World Health Organization recognizes nine types of erythroid dysplasia (Brunning *et al*, 2008). Although there are further categories and subcategories of abnormalities described on dysplastic bone marrow smears, concordance amongst expert haematologists reviewing identical dyserythropoietic slides is poor (Goasguen *et al*, 2018). Crookston's original description allows for a minority of dyserythropoiesis in normal bone marrow (Crookston *et al*, 1966).

There are three main types of CDA (CDA‐I, CDA‐II and CDA‐III) and although each has specific morphological and clinical features, blood films show overlapping abnormalities. All subtypes show anisocytosis and poikilocytosis and CDA‐I has macrocytic red cells while types II and III are usually normocytic (Bain *et al*, 2010). The major CDA subgroups were originally proposed based on specific erythroblast morphological abnormalities on bone marrow light microscopy of aspirates (Heimpel & Wendt, 1968): CDA‐I is indicated by binucleate macrocytic erythroblasts and internuclear bridging, CDA‐II by the presence of 10–35% binucleate late erythroblasts and CDA‐III by the presence of giant multinucleate erythroblasts with up to 12 nuclei per cell. Electron microscopy of erythroblasts reveals a characteristic pattern of chromatin abnormalities in CDA‐I (see below) and a double cellular membrane in CDA‐II. This classification system has facilitated the systematic collection of CDA cases, allowed the best available treatment to be delivered and led to the identification of causative genes. However, the degree to which these disorders share a molecular basis is unclear.

Since the discovery of *CDAN1* as a causative gene for CDA‐I in 2002, the genes for the three main types of CDA have been identified. Approximately 90% of CDA‐I cases are caused by bi‐allelic mutations in *CDAN1* or *C15orf41* (Dgany *et al*, 2002; Babbs *et al*, 2013), CDA‐II is caused by biallelic mutations in *SEC23B* (Schwarz *et al*, 2009) and CDA‐III is a dominant disorder caused by the P916R mutation of KIF23 (Liljeholm *et al*, 2013). Identification of the causative genes has shed light on the pathogenesis of these disorders, opened new avenues for research, allowed accurate molecular diagnosis and carrier testing of family members and impacted disease management. Variants of CDA have also been described, for example the dominantly inherited E325K mutation of *KLF1* causing an anaemia termed CDA‐IV and X‐linked forms caused by *GATA1* mutations (Nichols *et al*, 2000; Singleton *et al*, 2009; Arnaud *et al*, 2010). Further CDA subtypes have been suggested, however, the extent to which these are distinct entities will become clearer as our understanding of their molecular pathogenesis improves.

The abnormal cellular morphology and phenotypic abnormalities in CDA‐I are reviewed elsewhere (Wickramasinghe, 1997, 1998; Shalev *et al*, 2002; Heimpel, 2004; Tamary *et al*, 2005; Wickramasinghe & Wood, 2005; Heimpel *et al*, 2006, 2010; Iolascon & Delaunay, 2009; Renella & Wood, 2009; Iolascon *et al*, 2011, 2012, 2013; Gambale *et al*, 2016). Therefore, this review will focus on recent advances in the understanding of the diagnosis, management and pathogenesis of CDA‐I.

As with many rare disorders, establishing an accurate estimate of incidence and prevalence of CDA‐I is difficult. Over 300 cases have been reported (Iolascon *et al*, 2013). Most are sporadic cases from diverse regions such as Western Europe, North Africa and Asia, (Iolascon & Delaunay, 2009) while some series are accounted for by a founder effect, particularly in the Middle East (Tamary *et al*, 2005). The lack of reported cases from Sub‐Saharan Africa or South America may reflect ascertainment bias (Heimpel *et al*, 2010).

## Clinical presentation

**Figure 1:**
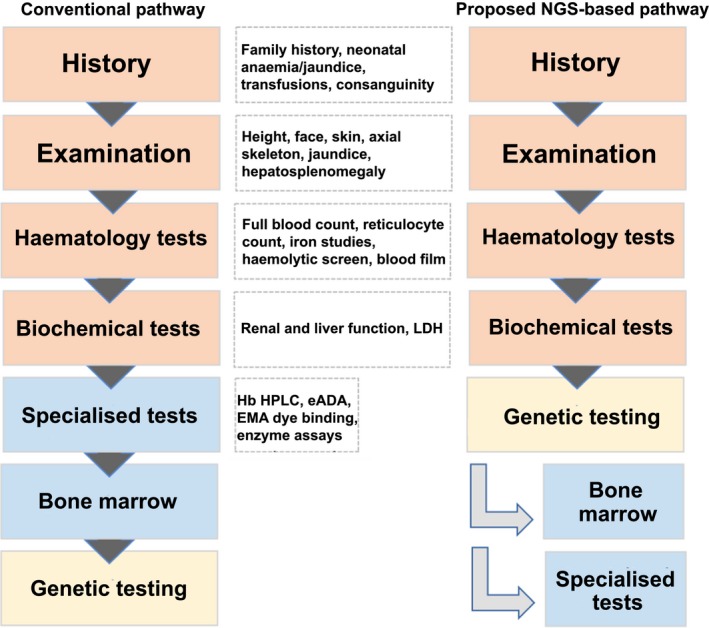
Comparison between the conventional pathway and an alternative NGS‐based pathway for the investigation of inherited anaemias. In the conventional pathway, patients are investigated sequentially where a differential diagnosis is devised based on history, examination and standard blood investigations. Specialised tests are then requested according to the suspected diagnosis and a bone marrow investigation frequently performed. Genetic testing is then reserved as a confirmatory test. In the alternative pathway, NGS is employed early, obviating the need for bone marrow biopsies in some patients where clear molecular diagnoses can be made by NGS. Where variants of uncertain significance are identified functional tests are required to confirm or refute the variant's pathogenicity. This alternative pathway can cut the time to diagnosis, remove the need for some bone marrow biopsies, provide accurate diagnosis of cases and allow genetic counselling. eADA, erythrocyte adenine deaminase; EMA, eosin‐5‐maleimide; Hb HPLC, haemoglobin high performance liquid chromatography; LDH, lactate dehydrogenase; NGS, next‐generation sequencing.

Diagnosis of CDA‐I is often predicated on a high index of suspicion. With rare disorders, awareness of the condition is necessary before appropriate investigations are instigated. Traditional pathways for the investigation of rare inherited anaemias follow the usual sequence of history and examination, standard haematological and biochemical assays, possibly radiological imaging, a bone marrow aspirate and trephine and genetic analysis. However, this approach is quickly changing with the advent of clinical‐grade next‐generation sequencing (Fig 1). Nevertheless, all investigations will be guided by a thorough initial consultation with the patient.

### History and examination

While some cases have been identified *in utero* (Parez *et al*, 2000; Kato *et al*, 2001; Lin *et al*, 2014), most present in childhood or young adulthood (Iolascon *et al*, 2011; Shalev *et al*, 2017). Cases detected *in utero* have led to fetal demise if untreated, but intra‐uterine transfusions support survival to term, followed by lifelong transfusion dependence. Later presentation can be due to intermittent jaundice and fatigue or the requirement for occasional blood transfusion for severe anaemia, while some have presented with secondary iron overload (Kawabata *et al*, 2012) or pigment gallstones (Fujino *et al*, 2013). Retrieving a neonatal history can be useful as some neonates with CDA‐I experience prolonged jaundice and/or the requirement for a blood transfusion perinatally, with no further transfusions thereafter. As a recessive disorder, CDA‐I is more likely to occur in consanguineous families and this should be directly questioned. Intriguingly, there is marked heterogeneity in phenotype expression, not only between unrelated patients with different mutations, but even in siblings with identical mutations, where, for example, one sibling presents in the neonatal period and the other at 2 years of age (al‐Fawaz & al‐Mashhadani, 1995; Heimpel *et al*, 2010).

Examination may reveal pallor, mild jaundice and splenomegaly, which is found in all cases, at least radiologically (Gambale *et al*, 2016; Shalev *et al*, 2017). The finding of frontal bossing can be observed in the more severe untreated cases, and this may prompt the need for imaging to assess for extramedullary haematopoiesis (Heimpel *et al*, 2003). The finding of limb abnormalities in the presence of anaemia should suggest CDA‐I.

### Haematology and biochemical assays

A full blood count typically reveals moderate macrocytic anaemia, with a haemoglobin (Hb) of 66–116 g/l (mean 92 g/l), mean corpuscular volume (MCV) 100–120 and reticulocytopenia, although 30% of cases have a normal MCV (Wickramasinghe, 1998). There are reports of transient neutropenia/thrombocytopenia, but these lineages are generally unaffected (Meznarich *et al*, 2018). The hallmark of CDA‐I is an absolute or relative reticulocytopenia, indicative of ineffective erythropoiesis (Heimpel, 2004). Ineffective erythropoiesis was originally demonstrated using ferrokinetic studies, where the fraction of ^59^Fe present in peripheral red blood cells is calculated 2 weeks post‐intravenous infusion. Where erythropoiesis is effective, e.g. when anaemia occurs due to bleeding, iron deficiency or haemolysis, the fraction of red cells containing ^59^Fe is ~75–80%, but in ineffective erythropoiesis, it may be as low as 25–30% (Lewis, 2001). Recently, a new clinical index, termed the bone marrow responsiveness index (BMRI), has been developed to discriminate haemolytic anaemia from ineffective erythropoiesis. This is defined as: [(absolute reticulocyte count) × (patient Hb/normal Hb)] and was shown to be a highly sensitive parameter (90·4%) to achieve a clinical diagnosis of CDA‐II. This metric is likely to be useful in CDA‐I but is yet to be formally validated in this disease.

**Figure 2:**
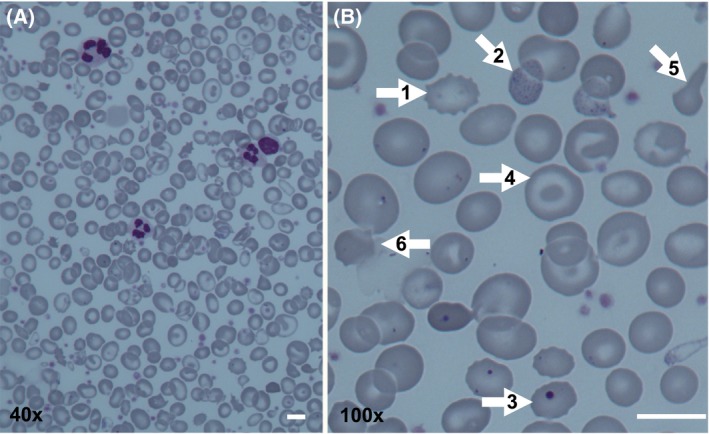
Peripheral blood film of a splenectomised patient with CDA‐I. (A) 40 × magnification, showing marked red cell anisopoikilocytosis. (B) 100 × magnification showing (1) echinocyte; (2) basophilic stippling; (3) Howell‐Jolly body; (4) target cell; (5) teardrop cell; (6) irregularly contracted red cell. Scale bars are 10 μm.

The blood film, as illustrated in Fig 2, is markedly abnormal with anisocytosis and various poikilocytes, including teardrops, ovalocytes, elliptocytes, microspherocytes, irregularly contracted red cells (Heimpel *et al*, 2010) and occasional nucleated red cells (Tamary & Dgany, 2009), although these are not a typical feature. Red cell distribution width, as a quantitative assessment of the aniosopoikilocytosis seen on films, is elevated in CDA‐I (Wickramasinghe, 1998; Kamiya & Manabe, 2010). Renal and liver function are normal unless perturbed secondary to severe iron overload, but unconjugated bilirubin and lactate dehydrogenase will be elevated from the haemolysis and ineffective erythropoiesis. Haptoglobin levels are reduced secondary to intra‐ and extra‐vascular haemolysis (Heimpel, 2004).

Key alternative diagnoses, which must be ruled out, include vitamin B12 and folate deficiency, autoimmune haemolytic anaemias, haemoglobinopathies or infections, such as human immunodeficiency virus or leishmaniasis. Ferritin and transferrin saturations should be assessed to evaluate for secondary iron overload. A high level of soluble transferrin receptor and low/unrecordable serum hepcidin in the absence of iron deficiency has also been noted in some CDA‐I patients (Cazzola *et al*, 1999), but neither test is readily available in the clinic.

### Bone marrow examination

**Figure 3:**
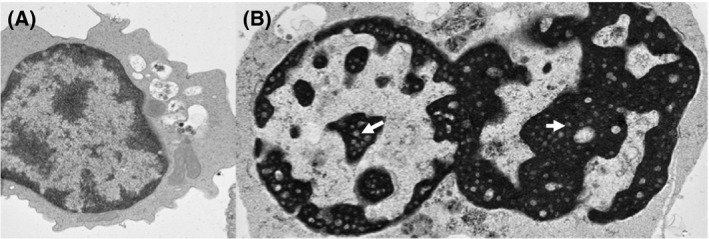
(A) Electron microscopy of a normal cultured erythroblast (day 7 of culture) showing the expected pattern of euchromatin and heterochromatin (magnification × 4000). (B) A CDA‐I cultured erythroblast showing binuclearity and the ‘Swiss cheese’ or ‘spongy’ heterochromatin with electron‐lucent areas (arrows) that is pathognomonic of this condition.

A bone marrow aspirate and trephine are usually carried out next, allowing rapid differentiation between, for example, Diamond Blackfan Anaemia (DBA) and CDA, both of which present with a reticulocytopenic anaemia. Findings on bone marrow examination were originally described by Heimpel and Wendt (1968); a hallmark of the condition is marked erythroid hyperplasia, with an excess of erythroid precursors compared to myeloid precursors (Heimpel, 2004). Between 2·4% and 10% of late polychromatic erythroblasts precursors are binucleate (Heimpel *et al*, 2010), where nuclei can be unequal sizes (Gambale *et al*, 2016). Intermediate erythroblasts exhibit internuclear bridges in 1–8% of cells examined and 30–60% of late erythroblasts exhibit a range of abnormalities, including polychromasia, megaloblastic changes, multinuclearity, karyorrhexis and basophilic stippling (Iolascon *et al*, 2012). At least 20% of erythroblasts must have an abnormality on light microscopy for a diagnosis of CDA‐I (Heimpel *et al*, 2010). The diagnosis is based on a constellation of abnormalities but the absence of internuclear bridges calls a diagnosis of CDA‐I into question. A recent study examining concordance in the morphological identification of dyserythropoiesis in two CDA‐I cases found agreement between only 4/7 expert haematologists (Goasguen *et al*, 2018). Diagnostic certainty relies on genetic confirmation or the gold standard of scanning electron microscopy (SEM) to identify nuclear abnormalities. This often requires a second bone marrow aspirate, as SEM samples require specific preparation for best results and CDA‐I is usually unsuspected prior to the light microscopy findings. SEM findings include widening of nuclear pores, darkening of heterochromatin with electron lucent areas within abnormally electron dense heterochromatin (Fig 3) described as ‘spongy heterochromatin’ (Wickramasinghe, 1998) or ‘Swiss cheese appearance’ (Heimpel *et al*, 2006). Invagination of the cytoplasm into the nucleus has also been described (Iolascon *et al*, 2013).

### Other features

Non‐haematological manifestations of CDA‐I are described in 10–20% of cases (Wickramasinghe, 1998), mostly involving the axial skeleton, such as missing distal phalanges, syndactyly (especially of the toes), and complete lack of nail formation (Brichard *et al*, 1994). Brown skin pigmentation (Heimpel & Wendt, 1968) and neurological deficits (Wickramasinghe, 1998) have occasionally been reported. However, as some families in which CDA‐I is diagnosed have a high degree of consanguinity, other features may represent epiphenotypes arising from different recessive conditions (Renella & Wood, 2009).

The presence of angioid streaks has been described in CDA‐I. These represent a break in Bruch's membrane, a collagenous layer underneath the pigment epithelium of the retina, which has been described in a small number of CDA‐I patients and is associated with loss of visual acuity (Frimmel & Kniestedt, 2016).

### Imaging

Abdominal ultrasound reveals universal splenomegaly (Heimpel *et al*, 2006) and gallstones in 50–60% of adults with CDA‐I (Shalev *et al*, 2002). Imaging with plain radiographs or magnetic resonance imaging (MRI) to investigate and characterise paraspinal masses secondary to extramedullary haematopoiesis may be necessary (Heimpel *et al*, 2009).

Imaging for organ iron overload by T2* MRI or Ferriscan^®^ is guided by biochemical iron studies. However, it has been reported that 38% of CDAs (type not specified) have cardiac iron on MRI, and 25% acquire cardiac iron loading by 10 years of age. Raised iron in the pancreas was described in six CDA cases and median liver iron concentration from T2* was 5·9 mg/g dry weight (upper limit of normal = 1·8 mg/g dry weight). While the role of imaging for iron loading is yet to be systematically evaluated in CDA‐I patients, this study suggests that this patient cohort warrants routine assessment of iron status (Berdoukas *et al*, 2013).

## Definitive diagnosis

### Genetic analysis

**Figure 4:**
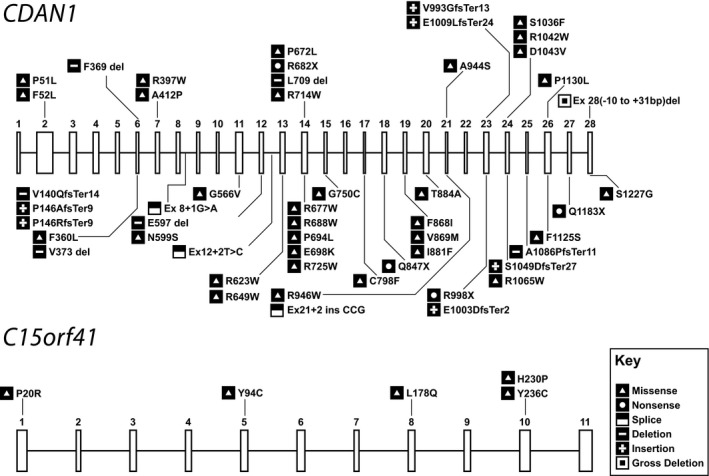
Representation of all known pathogenic variants associated with *CDAN1* and *C15orf41*. The exons in each gene are numbered to show the location of each mutation. Coding mutations are shown according to the amino acid change while splicing changes are shown according to their location relative to exons.

CDA‐I is a recessive disease caused by biallelic mutations in either *CDAN1* or *C15orf41* (Dgany *et al*, 2002; Babbs *et al*, 2013). There are 51 causative mutations documented in *CDAN1* and 5 in *C15orf41* (Fig 4). Currently no patients have been identified with compound mutations in these genes. Approximately 10% of CDA‐I cases remain unexplained by mutations in either of the known genes and there may be *cis*‐acting regulatory mutations affecting these genes or further, yet to be identified, loci causative of CDA‐I.

A recent review of the role of SEM in the diagnosis of CDA‐I concluded that genetic analysis for *CDAN1* and *C15orf41* should only be done once SEM has confirmed the presence of the pathognomonic ‘spongy heterochromatin’ abnormality (Resnitzky *et al*, 2017). We feel that genetic testing may obviate the need for SEM in some cases, thus avoiding an additional bone marrow aspirate with associated risks, which would be performed under general anaesthetic in the paediatric population. Genetic testing can be performed from a small amount of peripheral blood early in the diagnostic process (Fig 1). While knowledge of the precise underlying mutation does not currently carry prognostic information, it guides discussion, confirms the diagnosis and allows preimplantation genetic diagnosis.

Current approaches to genetic analysis include targeted panels (containing 50–200 genes), whole exome sequencing (WES) and whole genome sequencing (WGS). Where WES and WGS are undertaken for clinical diagnostics rather than research, e.g. as part of the National Health Service (NHS) England 100 000 genome project, the whole exome/genome is sequenced but only data from a pre‐determined set of genes is analysed. Targeted panels have been shown to be clinically useful in the diagnosis of rare inherited anaemias, including CDA‐I (Gerrard *et al*, 2013; Roy *et al*, 2016; Russo *et al*, 2018; Shefer Averbuch *et al*, 2018). Reported diagnostic rates vary from ~38% to ~65%, depending on the number and types of genes included and the depth of phenotypic assessment undertaken. In ~10% of cases, this approach reveals an unsuspected diagnosis (Roy *et al*, 2016). Making an accurate diagnosis is paramount to instituting correct therapy, for example steroids for DBA, splenectomy for pyruvate kinase deficiency and administration of interferon alpha (IFNa) for CDA‐I. In our recent study of a series of 20 cases with a presumed diagnosis of CDA‐I (from blood results and light microscopy and/or SEM), 55% received a molecular diagnosis from the targeted panel. The majority of these confirmed CDA‐I with mutations in *CDAN1* or *C15orf41*, but 20% had an alternative diagnosis, such as DBA with an unusual marrow in early childhood, and PK deficiency (Roy *et al*, 2016).

Extrapolating from these findings, up to 55% of patients may be able to avoid an unnecessary bone marrow aspiration when genetic analysis is performed earlier in the diagnostic pathway. In addition, time to diagnosis is notoriously long for patients with rare disorders and the delay between onset of symptoms and a formal diagnosis has been reported to be 12 years in some cases of CDA‐I (Fujino *et al*, 2013). A German CDA Registry reported the age of the 21 patients at the time of initial diagnosis of CDA‐I ranged between 0·1 and 45 years (median 17·3 years) and that 11 of 21 cases were previously misdiagnosed as congenital haemolytic anaemia **(**Heimpel *et al*, 2006). This underscores the utility of genetic testing to provide an accurate molecular diagnosis.

## Management

### Holistic approach

Akin to the management of thalassaemia patients, whose clinical course CDA‐I patients often closely resemble, the most favourable outcomes are achieved when CDA‐I patients are managed with a holistic and multidisciplinary approach. The diagnosis is usually a surprise to patients and their families and, as with rare disorders, patients suffer from a sense of isolation and that their physicians have inadequate knowledge about their condition (Budych *et al*, 2012). There is often the need for genetic counselling of the parents for future pregnancies. For patients receiving life‐long transfusions, issues such as long‐term intravenous access, logistics of regular transfusion and transfusion complications, are as important as they are in thalassaemia patients. Irrespective of transfusion status, CDA‐I is associated with iron overload and compliance with chelation is particularly important, especially around adolescence. Transition from paediatric to adult services should be carefully managed, and in the UK, National Institute for Health and Care Excellence (NICE) guidelines recommend the use of the “Ready, Steady, Go” approach (https://www.nice.org.uk/sharedlearning/implementing-transition-care-locally-and-nationally-using-the-ready-steady-go-programme). Complex patients may need to be managed by a team of psychologists, dietician, endocrinologists and bone specialists, while others will have a very mild disorder and require only a yearly review with a haematologist.

### Management of the anaemia

This depends on severity and patient characteristics and may change according to the patient's lifestyle. Some patients require transfusions only perinatally whilst others require them during additional marrow stress, e.g. intercurrent infections or pregnancy. True transfusion dependence occurs in 3–4% of cases (Heimpel *et al*, 2006), although genotype‐phenotype correlations have not yet been convincingly described. Transfusions increase the prevalence and severity of iron overload. Transfusion practice should follow guidelines for chronically transfused patients, and managing transfusion in CDA‐I patients according to practices developed for haemoglobinopathies is entirely appropriate. As such, blood units should be as fresh as possible, preferably <10 days since sampling from the donor (Milkins *et al*, 2013; Davis *et al*, 2017) and patients should receive the Hepatitis B vaccine and be offered lifelong folic acid replacement due to the haemolytic component. The only difference with haemoglobinopathy patients concerns the requirement for extended phenotyping to reduce the likelihood of developing antibodies. This is less critical than for the haemoglobinopathies where there is commonly a mismatch between the donor and recipient ethnicity (Davis *et al*, 2017), but is still considered best practice for CDA‐I patients.

### Interferon alpha

The only specific treatment available for CDA‐I is interferon alpha (IFNa). Its discovery was fortuitous when IFNa was given to a French patient with transfusion‐dependent CDA‐I who had contracted Hepatitis C through contaminated blood (Lavabre‐Bertrand *et al*, 1995) and became transfusion independent with reduction in ineffective erythropoiesis and amelioration of SEM features on bone marrow aspirate. Intriguingly, while 4 weeks of treatment ameliorated the erythroid:myeloid ratio and the SEM features reduced from 57% to 15·6%, the percentage of erythroblasts exhibiting internuclear bridges and binuclearity was unchanged. Prolonged treatment with IFNa leads to a stable Hb with ongoing normalisation in SEM features (Heimpel *et al*, 2006). Bilirubin falls in parallel as intramedullary haemolysis, characteristic of ineffective erythropoiesis, improves. Improvement in ineffective erythropoiesis in response to IFNa is supported by ferrokinetic studies (Lavabre‐Bertrand *et al*, 1995). Responses to IFNa are rapid, occurring within 4 weeks, but are not universal (Marwaha *et al*, 2005) and patients have required cessation of treatment due to side effects (Bader‐Meunier *et al*, 2005). These include gastrointestinal symptoms, flu‐like symptoms and depression. Reported effective doses vary between 4–10 million units per week (Heimpel, 2004; Lavabre‐Bertrand *et al*, 2004) but individual dose titration is required. Pegylated interferon at a dose of 30–50 μg/week has also been used effectively.

The mechanism of action of IFNa is not understood. *In vitro*, IFN production by Epstein–Barr virus‐transformed lymphocytes from CDA‐I patients is reduced, (Wickramasinghe *et al*, 1997) suggesting a deficit. It remains unclear whether CDA‐I patients respond to IFNa due to subnormal production of this *in vivo*, or whether the molecular defect leading to CDA‐I can be overcome by the over‐expression of IFN‐responsive genes. Further insights into the mechanism of action of IFNa may allow for the development of other, targeted therapies.

### Erythropoietin (Epo)

Epo levels are mildly elevated in CDA‐I patients, but remain inappropriately low for the degree of anaemia. Efforts to correct the anaemia using recombinant human Epo did not result in any rise in Hb, increase in reticulocyte count or fall in iron overload (Tamary *et al*, 1999). As such, Epo is not considered a treatment for CDA‐I.

### Splenectomy

Splenectomy in CDA‐I has not been evaluated systematically, but evidence from small case series suggests exercising caution. In one series, six patients were splenectomised for severe anaemia, five of whom had a rise in Hb (Shalev *et al*, 2017). However, this benefit was offset by a rise in mortality, with 3/6 patients dying between the ages of 40–60 from pulmonary hypertension (*n* = 1) and sepsis (*n* = 3). In another series, 7/21 patients underwent splenectomy with no response in Hb (Heimpel *et al*, 2006). Recent European Haematology Association recommendations highlight the high rate of complications in CDA‐I and suggest splenectomy should be reserved for patients with painful splenomegaly and/or significant thrombocytopenia/leucopenia (Iolascon *et al*, 2017).

### Management of iron overload

As detailed above, the aetiology of iron overload in non‐transfused CDA‐I patients is directly related to ineffective erythropoiesis. CDA‐I patients have unrecordable levels of hepcidin, leading to unopposed gastrointestinal iron absorption and deposition in target organs (Tamary *et al*, 2008; Kawabata *et al*, 2012). Suppression of hepcidin is hypothesized to result from excess erythroferrone production by the greatly expanded pool of erythroblasts. This mechanism has been successfully demonstrated in thalassaemia (Kautz *et al*, 2015) and CDA‐II (Russo *et al*, 2016). Serum growth differentiation factor 15 (GDF15) levels (a marker of ineffective erythropoiesis) were found to be elevated in CDA‐I patients, with a correlation between GDF15 and ferritin and anti‐correlation with serum hepcidin levels (Tamary *et al*, 2008).

There is no clinical consensus on the frequency of monitoring for tissue iron overload. In our centre we perform yearly T2* MRI in patients from the age of 10 years (Tamary & Dgany, 2009), although others investigate every 5 years once serum ferritin exceeds 600 μg/l from the age of 20 years (Gambale *et al*, 2016).

The management of iron overload in CDA‐I patients should be the same as for thalassaemia patients, namely chelators, be that sub‐cutaneous (deferrioxamine) or oral (deferiprone, deferasirox). However, a unique approach in these patients is to titrate the dose of IFNa such that the patient's Hb rises above that needed to avert the symptoms of anaemia so that regular venesections can be carried out. This has proven a very useful technique in some CDA‐I patients in clinical practice in several UK centres of which the authors are aware.

### Management of extramedullary haematopoieis

Extramedullary haematopoiesis (EMH), ranging in severity from minor to bulky, is a recognised complication of CDA‐I, although the exact prevalence is unclear (Heimpel *et al*, 2006, 2009). Management is in line with the EMH in thalassaemia with therapeutic options including surgical debulking, low dose irradiation and commencing regular transfusions to suppress EMH.

### Management of osteoporosis

Osteoporosis is present in 89% of CDA‐I cases (Shalev *et al*, 2017). The aetiology is probably multifactorial due to marrow expansion, diabetes and hypothyroidism, parathyroid gland dysfunction and the toxic effects of iron and chelators on osteoblasts (Voskaridou & Terpos, 2008). Active management entails treatment of calcium and vitamin D deficiency, bone densitometry scanning and regular review by bone specialists (Shalev *et al*, 2017).

### Management of pregnancy

Shalev *et al* (2004) reviewed the outcomes in 28 spontaneous pregnancies in 18 women over a 15‐year period in a Bedouin tribe. The complication rate was high (64%) and included one first trimester spontaneous abortion, one stillbirth and 42% low birth weight infants. The rate of infants requiring caesarean sections was statistically significantly higher than a control group of Bedouin women (35·7% vs. 11%) for reasons including fetal distress and pre‐eclampsia. Not infrequently, previously transfusion independent women with CDA‐I develop a transfusion requirement during pregnancy (Roy & Pavord, 2018).

### Management of endocrinopathies

Endocrinopathies have been described in 10–40% of CDA‐I patients and include diabetes mellitus and hypothyroidism (Heimpel *et al*, 2006; Shalev *et al*, 2017), as well as pituitary failure leading to growth retardation (Facon *et al*, 1990). These are thought to be secondary complications from iron overload and poor compliance with chelation. Their management requires close collaboration with endocrinologists as well as psychologists to aid medication compliance.

### Bone marrow transplantation

Paediatric bone marrow transplants have been carried out in a few patients with CDA‐I. In a small series, three children underwent matched sibling allografts. All were severe and diagnosed before 6 months of age, had hepatosplenomegaly and two required chelation. Conditioning was with cyclophosphamide 50 mg/kg/day for 4 days, busulfan 4 mg/kg/day for 4 days and antithymocyte globulin 30 mg/kg for 4 doses pre‐stem cell transplantation. All three engrafted and became transfusion independent (Ayas *et al*, 2002). One of the barriers to transplantation is poor prognosis conferred by pre‐transplant iron overload, yet the most severe patients who would most benefit from transplant are the ones whose response to chelation is limited (Buchbinder *et al*, 2012).

### Pulmonary hypertension

CDA‐I may present with pulmonary hypertension in the neonatal period, in association with other congenital anomalies (El‐Sheikh *et al*, 2014; Landau *et al*, 2015). Treatment includes inhaled nitric oxide and high frequency oscillation ventilation. While pulmonary hypertension may be a late complication of CDA‐I, the extent of this is unknown. There are currently no guidelines on whether and how pulmonary hypertension should be assessed and treated in CDA‐I patients. For patients with sickle cell disease, this is screened by tricuspid jet velocity followed by right heart catheterization in patients with tricuspid jet velocity >2·5 m/s. A recent meta‐analysis (Wang *et al*, 2018) compared different types of treatment for all types of idiopathic pulmonary hypertension including endothelin receptor antagonists, phosphodiesterase type‐5 inhibitors, prostaglandin I2, soluble guanylate cyclase stimulator and selective non‐prostanoid prostacyclin receptor agonists or combination treatment. Results differed depending on the outcome used to measure response but the best responses appeared to be for vardenafil (taken orally) and iloprost + bosantan (inhaled). Whether these would be the best treatment for CDA‐I patients with pulmonary hypertension is not known.

### Potential for gene editing

Gene editing could provide hope for a cure. In CDA‐I, gene editing would require homology‐directed repair to integrate a donor template and so each targeted mutation would require individually designed editing, which remains currently out of reach. However, in the longer term, elucidation of the pathway affected in CDA‐I may identify a therapeutic target gene, which would allow a universal therapy to be developed.

## Pathogenesis

Rare diseases (defined as those affecting <1:2000 of the population) have been estimated to number between 6000–8000, collectively affecting some 30 million European Union citizens (http://www.eurordis.org/). However, the study of rare diseases has an impact reaching far beyond affected individuals alone: for example, research into the molecular basis behind Fanconi anaemia, a congenital bone marrow failure syndrome affecting ~1000 individuals worldwide, has provided critical insights into the link between genomic instability and malignancy, has significantly advanced our understanding of DNA repair mechanisms (Schindler & Hoehn, 2007 *)* and has led directly to the development of therapeutic agents for patients with *BRCA1*/*2* mutations (Fong *et al*, 2009).

**Figure 5:**
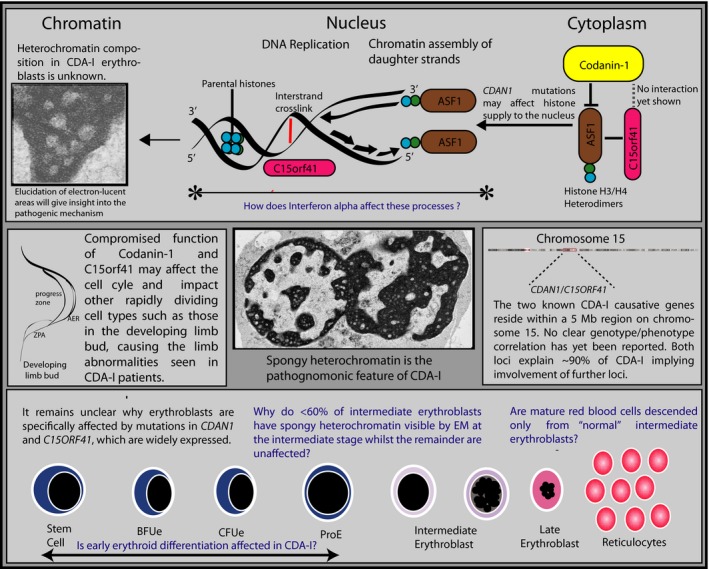
Summary of current knowledge of pathophysiology of CDA‐I (black type) and key questions (blue type). The top panel focuses on different sub‐cellular compartments. While spongy heterochromatin is the pathognomonic feature of CDA‐I, the composition of the electron‐lucent areas is unknown. Whether they are true euchromatin or abnormally packaged heterochromatin would indicate the function(s) of the key proteins. The known interactions between Codanin‐1, C15orf41 and the histone chaperone ASF1 are shown and the abnormal shuttling of these proteins into the nucleus in the context of mutated CDAN1 in non‐erythroid cells suggests a possible problem in delivery of histones in the rechromatinisation of replicating DNA. Because of its predicted role as a nuclease, C15orf41 may play a role in clearing blocks to replication fork progression (such as interstrand cross‐links) or replication intermediaries. Determining whether interferon alpha acts at this level would narrow down the key cellular processes in which Codanin‐1/C15orf41 are involved. In the middle left panel, the possibility that Codanin‐1 has a direct function in the developing limb bud by affecting the coordinated interaction between the signalling centres is suggested. In the right middle panel the chromosomal location of CDAN1 and C15orf41 is shown and the existence of further loci is strongly suggested by the lack of molecular diagnoses in 10% of EM‐proven CDA‐I cases. The bottom panel illustrates erythroid differentiation and the conundrums of the predominantly erythroid abnormalities in CDA‐I despite broad expression of *CDAN1* and *C15orf41* and the fact that only subset of erythroid progenitors are morphologically affected in the bone marrow. AER, apical ectodermal ridge; Stem cell, haematopoietic stem cell; BFUe, burst forming unit (erythroid); CFUe, colony forming unit (erythroid); ProE, pro‐erythroblast; ZPA, zone of polarising activity.

CDA‐I is an example of a rare disease which has the potential to inform about general cellular processes. Codanin‐1 (CDAN1) and C15orf41 expression levels are extremely low in all cell types, yet are widely expressed, and loss of either protein is incompatible with life. Both proteins are likely to play a critical role in DNA repair and/or chromatin assembly following DNA replication and understanding their function will elucidate universal cellular processes. A number of fundamental questions remain unanswered about the biology and pathology of CDA‐I (Fig 5). The main hurdle to advancing our understanding of the function of CDA‐I proteins is the lack of molecular reagents, including antibodies, appropriate cell lines and access to primary material, although attempts have been made to address this by generating erythroid cell lines with engineered tags at the endogenous loci (Moir‐Meyer *et al*, 2018).

## Gene function

Much of our knowledge about Codanin‐1 and C15orf41 derives from studies in osteosarcoma (U‐2‐OS) cells and cervical cancer (HeLa) cells, both of which are cytogenetically abnormal cell lines that may not reflect biology in primary erythroid cells. For example, Codanin‐1 knock‐down in U‐2‐OS cells results in a faster cell cycle (Ask *et al*, 2012), whereas deletion of the endogenous *Cdan1* in mice results in early embryonic lethality (Renella *et al*, 2011). Both CDA‐I proteins are widely expressed and yet, when mutated, affect only the erythroid lineage, suggesting the pathological mechanism must be investigated in this cell type. However, insights into potential functions of these proteins gained from studies in non‐erythroid cells will be presented.

### Codanin‐1

Bi‐allelic mutations of *CDAN1* account for ~80% of CDA‐I cases. The gene comprises 28 exons and encodes the protein Codanin‐1, which is relatively large (~134 kD) and highly evolutionarily conserved in fish, frogs and flies with no human orthologs and no apparent homologue in worms and yeast (Dgany *et al*, 2002). The *Drosophila* homologue, discs lost (Dlt), is required for cell survival and cell‐cycle progression (Pielage *et al*, 2003). There are no functionally conserved domains in Codanin‐1 to facilitate functional predictions, however, a putative peptide binding site has been identified through which Codanin‐1 may interact with the well‐described histone chaperone Asf1 (Tamary *et al*, 2010; Ask *et al*, 2012).

Regulation of *CDAN1* expression appears to depend on the cell cycle as the promoter contains several binding sites for the cell‐cycle regulated transcription factor E2F that increases expression of Codanin‐1 in S‐phase in HeLa cells (Noy‐Lotan *et al*, 2009). Codanin‐1 is reported to be enriched in the nucleus in the K562 erythroleukaemia line, U‐2‐OS and HeLa cells. Codanin‐1 has been found to associate with DNA during interphase in HeLa cells and excluded from mitotic condensing chromosomes (Noy‐Lotan *et al*, 2009). However, other reports suggest that Codanin‐1 is mainly localised to the cytoplasm in U‐2‐OS cells (Ask *et al*, 2012). This discrepancy needs to be resolved by determining the localisation in primary erythroid cells using independent antibodies when the protein is expressed at native levels.

### C15orf41

Bi‐allelic mutations in *C15orf41* cause ~10% of CDA‐I cases (Babbs *et al*, 2013). Similar to Codanin‐1, C15orf41 protein is widely conserved with orthologs broadly distributed in eukaryotes and in members of the archaea and viruses, indicating a highly conserved function. *C15orf41* is present in all species where *CDAN1* is found, and none where it is not, suggesting the proteins function in concert. Additionally, the pathognomonic CDA‐I heterochromatin defects that arise when either gene is mutated strongly suggest a common pathway, although a direct interaction between the two proteins has not been shown (Fig 5). Sequence conservation shows C15orf41 protein belongs to the PD‐(D/E)XK family of restriction endonucleases, a diverse group of phosphodiesterases involved in genome maintenance processes, such as DNA damage repair, Holliday junction resolution and RNA processing (Laganeckas *et al*, 2011). Notable members of this family include the DNA repair nucleases Mus81 and XPF (ERCC4/FANCQ) that play key roles in DNA lesion resolution and maintenance of genome stability (Steczkiewicz *et al*, 2012). This points to a defect in DNA repair in CDA‐I, however, the nature of the lesions that result from impaired C15orf41 function remain unknown.

### Role in chromatin assembly

C15orf41 and Codanin‐1 both interact with the histone chaperone Asf1b (anti silencing factor 1b) (Ewing *et al*, 2007; Ask *et al*, 2012). Asf1 is essential for chromatin assembly in human cells (Groth *et al*, 2005, 2007), playing a role donating new histones to chromatin assembly factor 1 (CAF1) (Mello *et al*, 2002) for incorporation into nucleosomes following DNA replication. Asf1 binds histone H3‐H4 heterodimers in the cytoplasm and chaperones them into the nucleus where they are transferred to downstream chromatin assembly factors (Campos *et al*, 2010; Jasencakova *et al*, 2010).

Codanin‐1 sequesters Asf1 in the cytoplasm, thereby negatively regulating the supply of histone bound Asf1 to the nucleus (Ask *et al*, 2012). Mutations in Codanin‐1, which impair the interaction with Asf1, may allow unregulated Asf1 to access the nucleus thereby disrupting the fine‐tuned delivery of histones known to be critical in correctly rechromatinising newly synthesised DNA (Jasencakova & Groth, 2010). It remains to be shown to what extent abnormal histone delivery at the replication fork leads to the specific abnormalities in chromatin and heterochromatin seen in CDA‐I erythroblasts.

### Lineage specificity

*CDAN1* and *C15orf41* are ubiquitously expressed, albeit at a relatively low level in most tissues, and no individual harbouring two loss of function alleles of either gene has been identified. Additionally, mice embryos homozygous for null *Cdan1* alleles die prior to implantation, suggesting that Codanin‐1 is essential prior to the onset of erythropoiesis (Renella *et al*, 2011). Given that Codanin‐1 and C15orf41 are highly conserved, ubiquitously expressed and appear essential, it is of great interest that abnormalities in CDA‐I are restricted to the erythroid lineage, suggesting that erythroblasts have a specific requirement for Codanin‐1 and C15orf41.

One possibility may be that erythroid progenitors have a uniquely fast cell cycle, although CDA patients do not manifest abnormalities of other tissues containing fast‐dividing cell types, such as gut epithelium or hair follicles. It has been reported that, in mice, a particularly rapid cell cycle is required at the start of terminal erythroid differentiation for erythroid lineage commitment, through a mechanism of passive genome demethylation causing the PU.1 (also termed SPI1) switch and lineage commitment (Pop *et al*, 2010; Shearstone *et al*, 2011). However, it remains to be shown whether a similar phenomenon exists in human erythroid differentiation and how this may be impacted by impaired chromatin assembly. Other hypotheses include nuclear extrusion in erythroblasts, which requires the eviction of histones such as H3 and H4 (Zhao *et al*, 2016) and C15orf41 and Codanin‐1 may play a role in this process. It also may be of significance that the proportion of erythroblasts displaying chromatin abnormalities varies from patient to patient and remaining erythroblasts appear to undergo normal terminal maturation, suggesting a threshold effect.

Whether the non‐haematological manifestations of CDA‐I reflect severe anaemia *in utero* or are directly due to the effects of a mutated or reduced amount of Codanin‐1, as has been previously suggested (Goede *et al*, 2006), is difficult to ascertain. Certainly, some features, such as diabetes and growth retardation are more readily ascribed to the iron overload that accompanies CDA‐I. However, the ubiquitous expression of Codanin‐1 and C15orf41 in all tissue types suggests a direct effect in tissues beyond the erythroid lineage. Compromised cell division may affect the rapidly dividing cells of the progress zone and the apical ectodermal ridge in developing limb buds (Tickle, 2015). Limb abnormalities in CDA‐I patients are usually asymmetrical reductions, suggesting a defined signalling pathway is not uniformly affected. However, compromised cell division is likely to be stochastic with a threshold effect, as seen in erythroblasts, and therefore malformations would not be expected to be uniform or bilateral. In addition, if the skeletal defects were due to tissue hypoxia, they could be expected to mirror those seen in Bart's Hydrops Fetalis Syndrome where the absence of HbF creates severe hypoxia. However, in that form of thalassaemia non‐haematological manifestations are more neurological and urogenital, with no descriptions of acral dysostosis (Songdej *et al*, 2017).

## Erythroblast abnormalities

Characterisation of the cellular abnormalities in CDA‐I erythroblasts could shed light on the function of the two genes involved. Analysis of cell cycle distribution in cultured erythroblasts showed an increase in cells in S‐phase in CDA‐I, which are, paradoxically, not actively synthesising DNA when tested, suggesting an S‐phase arrest in CDA‐I (Wickramasinghe & Pippard, 1986). This points to a problem with DNA replication, but needs to be refined using a larger number of patients. Unrepaired DNA lesions act as physical barriers to replication fork progression and nicks, gaps and stretches of ssDNA can be both sources and symptoms of stress (Zeman & Cimprich, 2014). Because C15orf41 is predicted to be an endonuclease it may be that there are more unrepaired lesions in CDA‐I patients and therefore more stalled replication forks, underlying the proposed S‐phase arrest. Alternatively, replication intermediaries that are usually cleared by C15orf41 may underlie some of the nuclear abnormalities seen in CDA‐I erythroblasts. Given that these events are stochastic and would also be likely to be affected by genetic background, this may go some way to explaining the variability between patients. It would be very informative to distinguish these possibilities (blocked replication vs. unresolved replication intermediates).

Elucidating the nature of the electron‐lucent areas in the heterochromatin seen by SEM (Fig 3) would shed light on the underlying pathology of CDA‐I, especially as resolving this abnormality is associated with improved Hb levels in patients treated with IFNa (Lavabre‐Bertrand *et al*, 1995, 2004). The differentiating stain used in SEM is osmium tetroxide, however, why this differentially binds the heterochromatin in affected nuclei is unclear. It may be that the “holes” contain protein, lipids or improperly packaged heterochromatin affecting its transcriptional status.

Do the mutations in either *CDAN1* or *C15orf41* represent different entities? It has been suggested that CDA‐I arising from mutations of *C15orf41* may be more severe (Gambale *et al*, 2016). In our clinical and laboratory experience, at the time of writing there are insufficient data to draw this distinction with certainty. Given that CDA‐I with specific chromatin abnormalities arises from mutation of either gene, we propose that this disease simply be termed CDA‐I until new insights on genotype/phenotype correlations are obtained.

## Conclusions

### Patient welfare

CDA‐I remains a rare disorder and shares some of the hurdles and obstacles borne by patients with other rare conditions. Delays in diagnosis will hopefully be reduced by the implementation of gene panels as part of routine testing. Earlier diagnosis should allow treatment prior to the development of iron overload and associated organ damage. Due to the rarity of the condition, CDA‐I patients should be reviewed at least annually in a centre with a specialist interest in rare anaemias and access to specialized monitoring and multidisciplinary meetings. Such clinicians belong to national and international networks of experts which collaborate to provide optimal care and access to research developments. EuroBloodNet, a European rare disease network, promotes trans‐national working and sharing of expertise. Finally, patients with rare conditions benefit from support offered by national patient networks. A recent James Lind Alliance Priority Setting Partnership identified the number one (of ten) research questions important to these patients and their families as “*Would a national formal network of clinicians with expertise and/or a national MDT (multidisciplinary team meeting) improve care for patients with rare inherited anaemias?”* (http://www.jla.nihr.ac.uk/priority-setting-partnerships/rare-inherited-anaemias/top-10-priorities.htm). Therefore, collaboration between clinicians, patients and research partners is critical to guiding and securing optimal clinical care for CDA‐I.

## Author Contributions

NR and CB conducted the literature review and wrote the manuscript.
